# The relationship between facility-based malaria test positivity rate and community-based parasite prevalence

**DOI:** 10.1371/journal.pone.0240058

**Published:** 2020-10-07

**Authors:** Alice Kamau, Grace Mtanje, Christine Mataza, Lucas Malla, Philip Bejon, Robert W. Snow

**Affiliations:** 1 KEMRI-Wellcome Trust Research Programme, Kilifi, Kenya; 2 Centre for Tropical Medicine and Global Health, Nuffield Department of Clinical Medicine, University of Oxford, Oxford, United Kingdom; 3 Ministry of Health, Kilifi County Government, Kilifi, Kenya; Universidade Federal de Minas Gerais, BRAZIL

## Abstract

**Introduction:**

Malaria surveillance is a key pillar in the control of malaria in Africa. The value of using routinely collected data from health facilities to define malaria risk at community levels remains poorly defined.

**Methods:**

Four cross-sectional parasite prevalence surveys were undertaken among residents at 36 enumeration zones in Kilifi county on the Kenyan coast and temporally and spatially matched to fever surveillance at 6 health facilities serving the same communities over 12 months. The age-structured functional form of the relationship between test positivity rate (TPR) and community-based parasite prevalence (PR) was explored through the development of regression models fitted by alternating the linear, exponential and polynomial terms for PR. The predictive ranges of TPR were explored for PR endemicity risk groups of control programmatic value using cut-offs of low (PR <5%) and high (PR ≥ 30%) transmission intensity.

**Results:**

Among 28,134 febrile patients encountered for malaria diagnostic testing in the health facilities, 12,143 (43.2%: 95% CI: 42.6%, 43.7%) were positive. The overall community PR was 9.9% (95% CI: 9.2%, 10.7%) among 6,479 participants tested for malaria. The polynomial model was the best fitting model for the data that described the algebraic relationship between TPR and PR. In this setting, a TPR of ≥ 49% in all age groups corresponded to an age-standardized PR of ≥ 30%, while a TPR of < 40% corresponded to an age-standardized PR of < 5%.

**Conclusion:**

A non-linear relationship was observed between the relative change in TPR and changes in the PR, which is likely to have important implications for malaria surveillance programs, especially at the extremes of transmission. However, larger, more spatially diverse data series using routinely collected TPR data matched to community-based infection prevalence data are required to explore the more practical implications of using TPR as a replacement for community PR.

## Introduction

Monitoring the intensity of malaria transmission in time and space is an important parameter to define the required combinations of intervention to accelerate control and elimination, measure impact and over time repurpose intervention and control ambitions [1–3].

The prevalence of malaria infection among community residents, or school attendees, has been used for over a century as a marker of the quantity of malaria (endemicity) in a location [4, 5], and is often the key metric used in malaria indicator surveys by National Malaria Control Programmes (NMCPs). Recently, community prevalence has been used as part of geostatistical-epidemiological models to estimate the burden of malaria in sub-Saharan Africa (SSA) [3, 6, 7] and to predict future scenarios of malaria control [8]. During the most recent attempt to model age-standardised (2–10 years) *Plasmodium falciparum* prevalence (*Pf*PR_2–10_), 43,187 empirical survey estimates were used to provide 16,200,000 5 × 5 km predictions of malaria prevalence, disease and mortality in stable endemic areas of Africa between 2000 and 2017 [7].

The sparsity of community-based prevalence surveys remains an important source of error in the predictions of malaria endemicity and disease burden in time and space across SSA [9]. National household surveys are undertaken among small, randomly selected clusters aimed to be representative of large sub-national administrative units and surveys are powered on bed net use rather than infection prevalence [10, 11]. These surveys occur infrequently, every 3–5 years; some SSA countries have not undertaken a national survey since 2000 (Niger, Mauritania, Peoples Republic of Congo and Central African Republic). A few countries have augmented household surveys with parasitological surveys among school children [12–18]. These cross-sectional surveys are undertaken at one single time point, and several national household surveys have been conducted during non-malaria seasons for logistical reasons. With a few exceptions (Sudan, Somalia, Djibouti, and Kenya) household surveys have focussed on measuring malaria infection in young children aged 6 months to five years. Under-fives represent an important target for intervention coverage as they bear the brunt of the disease burden. However, infection prevalence as a marker of endemicity is better described in children aged 2–10 years [4, 19]. Household surveys are logistically demanding and expensive; for example, in Tanzania, the average cost of a recent school survey was US$ 10 per subject examined compared to US$ 410 per subject for the household survey [14].

A more continuous and spatially ubiquitous source of information is the prevalence of malaria infection among patients examined in health facilities. Traditionally, this was referred to as slide positivity rate and was recommended as a transmission surveillance metric when community-based prevalence fell below 2%; a point where community sampling became financially impractical [1, 20, 21]. With the rolling out of the WHO’s policy on test-treat-track [22], the universal acceptance and delivery of malaria rapid diagnostic tests (mRDTs) across Africa [3] and the adoption of digital health data capture platform (DHIS2) [23], opportunities exist to use test positivity rates (TPR) as a measurable, temporal quantity of malaria endemicity in more localities across Africa than provided by community prevalence surveys [2, 24].

While the detection of all malaria cases represents a core intervention for elimination [25], the use of routine health facility TPR data for malaria endemicity stratification and surveillance at national scales remains underutilised in the high burden areas of SSA due to their imperfections [23]. Several examples of the use of routine TPR data exist to understand the sub-national variations in malaria risk [26–30], at smaller spatial scales to define temporal trends [31–37], or intervention impact [38–41]. However, there have been few direct comparisons between community-based parasite prevalence and facility-based TPR [24, 42, 43]. Here the relationship between time-space matched TPR at health facilities and community-based prevalence of infection on the Kenyan coast was examined.

## Materials and methods

### Study area

**Fig 1:**
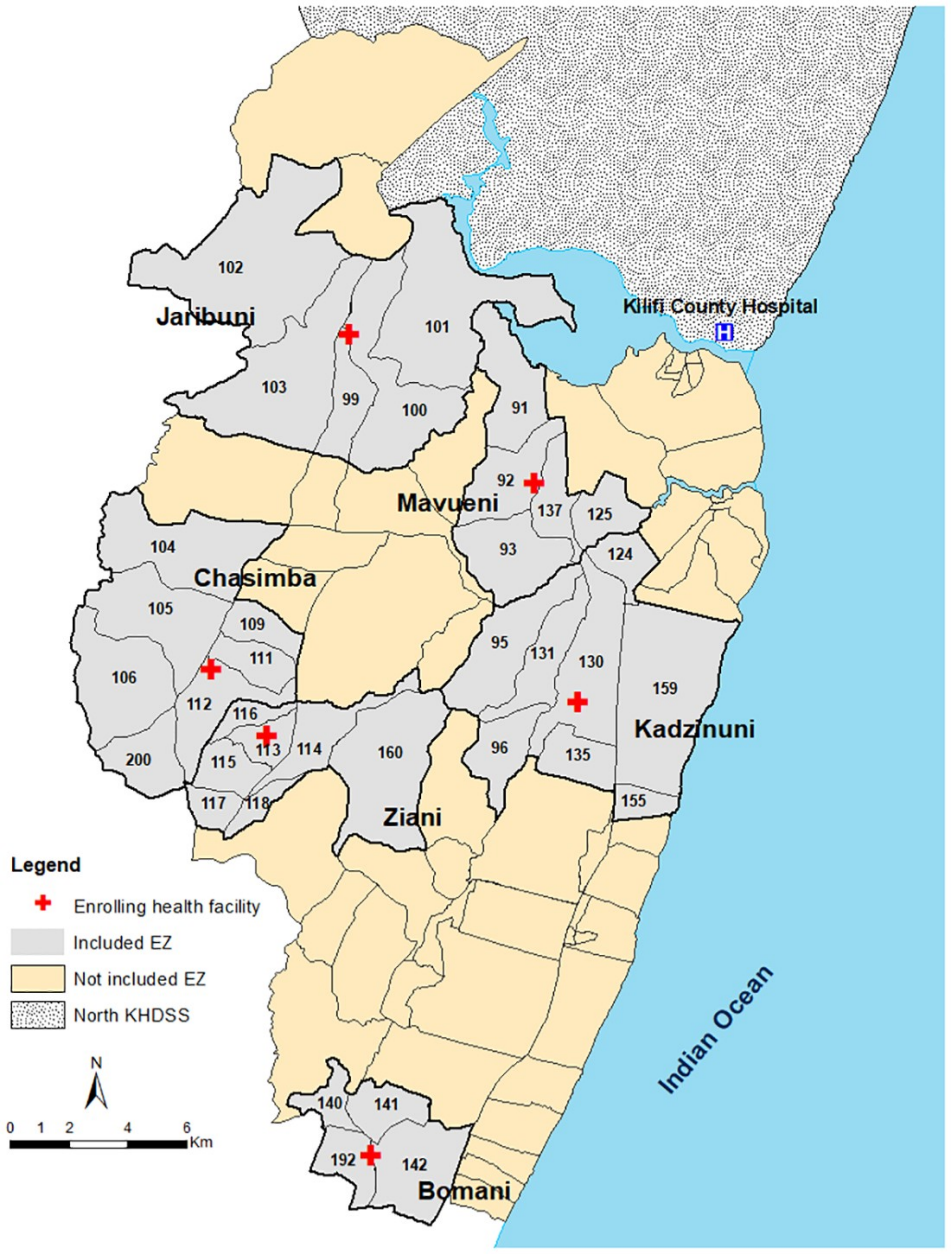
Map showing the location of the study sites and included enumeration zones (EZs).

This study was conducted in the southern part of Kilifi Health and Demographic Surveillance System (KHDSS) located along the Kenyan coast [44]. The study locations have been described in detail elsewhere [45]. Malaria transmission in this area is supported predominantly by *Anopheles gambiae s*.*l* and *An*. *funestus* s.s. [46, 47] and follows a bi-modal pattern associated with the long (April-June) and short (October-December) rains. Six public health facilities providing curative services were selected and the KHDSS enumeration zones (EZ) surrounding each facility within a 2 km radius (Fig 1). The area included 36 EZs consisting of 9,596 homesteads and an enumerated mid-year population of 72,560 in 2018. The six health facilities were selected on the basis that they were public health facilities and were more likely to comply with government policies on diagnosis, treatment and participate in routine reporting of data. They also had a high burden of patients (a minimum of 10 patients per day), and were not part of ongoing active surveillance. During the surveillance period, 84% of children under-five years slept under insecticide treated nets.

### Study procedures

The study in the health facilities was established as a partnership with the County Ministry of Health and was developed to reflect routine practices as far as was possible. The national standard treatment guidelines for malaria in Kenya specify that all patients presenting with fever should be investigated parasitologically [48] and the current information system mandates recording of malaria rapid diagnostic test (mRDT) results. The study aimed to ensure that all patients of all ages presenting with fever were tested and that all information was documented [48]. At each facility, the study involved records collected using a study form for patients that sought treatment between March 2018 and February 2019. For a patient to be included in the study they had to be ≥ 6 months of age with a history of fever in the last 24 hours as part of presenting illness or a measured axillary temperature ≥37.5°C, hereafter referred as febrile patients. All febrile patients were tested using a malaria rapid diagnostic test (mRDT) (CareStart^™^) to detect HRP2 specific to *P*. *falciparum*. If the mRDT results were positive the patient received appropriate treatment as per the Government of Kenya guidelines for malaria-case management [48]. HRP2 based mRDTs continue to have acceptable sensitivity and specificity in coastal Kenya [49]. The patient’s residence was documented and matched to the enumerated KHDSS geo-coded homestead register.

Four community-based prevalence surveys were undertaken during the facility surveillance period; May–June 2018, August 2018, October 2018 and December 2018– January 2019. During each survey round, random homesteads were selected and in the subsequent sampling frames, previously selected homesteads were excluded. A sample size of at least 4,341 participants was obtained based on the local prevalence estimated to be ≤ 30% [50], a precision of 1.5% and an expected refusal rate of 5%. The participants were frequency-matched to cases in the health facility by season and age group. The surveillance of infection prevalence in the community therefore required a minimum of 4 participants in each of the 60 randomly selected homesteads in the catchment area of the six health facilities during each survey round. For a participant to be included in the study they had to be ≥ 6 months of age, residents of the catchment area of the facility, and had agreed to participate in the study. Participants that did not fulfil the three criteria were excluded. For each consenting homestead member aged ≥ 6 months, the fieldworkers obtained information on the participant’s demographics. Fever was assessed as an axillary temperature ≥ 37.5°C; or a history of reported fever in the last 24-hours. A malaria test was performed on all consenting participants using mRDT (CareStart^™^), irrespective of their fever status. All participants with fever and/or a positive rapid test were advised to seek treatment at the nearest health facility.

Data was entered electronically using laptops in the facilities and tablets in the community-based surveys by the study team on a PHP web-based interface and data saved onto MySQL database and synchronized onto a secure server.

### Data analysis

Analysis included only data for participants identified as residents of the catchment areas shown in Fig 1. Pregnant women and participants that had been enrolled in either the health facility or community survey within the last 14 days were excluded. Descriptive statistics included proportions with 95% confidence intervals (CI), means with standard deviation (SD), and medians with interquartile range (IQR). A Chi-square test was used to compare difference in proportions.

The health facility TPR was defined as the number of positive diagnostic tests as a proportion of the total tests performed among febrile patients. The community parasite prevalence (PR) was defined as the proportion of participants tested found with a positive mRDT. To compute the TPR and PR for each EZ, the number of positive participants and total tests performed were aggregated at the EZ level. For each EZ, the facility TPR data were matched to two-month period around each community-based cross-sectional survey. To explore different temporal matches, the time interval was altered by 1) matching the health facility data to the exact same four-time periods when the community-based survey was conducted; 2) matching to the subsequent month i.e. lagging the health facility data by one month after the community cross-sectional survey; or 3) by using all the health facility data in each EZ versus all four community-based prevalence surveys in each EZ. TPR and PR values were compared using actual age-groupings and against the traditional PR_2–10 years_ using data from the entire community age-standardised to the 2–10 age group in each EZ as described elsewhere [19]. The association between health facilities TPR and PR (age-matched and age standardized 2–10 years) was determined using Spearman’s rank correlation; and the varying facility time-periods used as a sensitivity analysis.

To explore the functional form of the relationship between TPR and PR in a more formal form, i.e. to define a function (F), that transforms an estimate of PR over any range (x, y), into a TPR over any range (L, U), i.e. F: PR(x, y)→TPR(L, U), various models were considered. The relationship was assessed using a linear relationship and other flexible function forms using the polynomial and exponential transformations. A selection of the best fitting model was made using goodness of fit, measured as root mean square error (RMSE), adjusted R^2^, and Akaike Information Criteria (AIC). Regression diagnostics were performed including Cook’s D to assess for high leverage, which was indicative of influential EZ, and/or large residuals (outliers). To test the variability in the prediction performance of the models, 80% of the data were divided into train and 20% into test datasets, and the model was rerun on 100 randomly selected samples. The models developed by the training dataset were validated against the test datasets by comparing the accuracy measures i.e. the correlation between actual and predicted estimates and the error rates (mean absolute percentage error (MAPE), mean square error (MSE) and mean absolute error (MAE)). As a sensitivity analysis, the effects of using varying intervals of TPR in quantifying the relationship between TPR and PR using the best fitting model was explored.

Finally, the predictive ranges of TPR were explored for PR endemicity risk groups of programmatic value, i.e. cut-offs of low (PR <5%) and high (PR ≥ 30%), used in national malaria stratification in Kenya [50–52] and Tanzania [30]. To serve as a guide to set the appropriate cut-offs for low transmission settings, the upper bound of the 95% confidence interval (CI) of the predictive range of TPR was used as a conservative measure. While the lower bound of the 95% CI of the predictive range of TPR was used in high transmission. Data analysis was performed in Stata, version 13 (Stata Corporation, College Station, TX) and R version 3.6.1 (R Core Team (2019), Vienna, Austria). ArcMap version 10·5 (ESRI Inc., Redlands, CA, USA) was used to develop the map in Fig 1. The shapefiles were downloaded from an open source website (http://www.diva-gis.org/gdata).

The health facility surveillance did not impose any changes in the national treatment guidelines and data used in the analysis were gathered as part of routine care. Consent was waived by the ethics committee; therefore, individual patient consent was not sought. All the records were pseudo-anonymized at the point of data capture in the healthcare facilities, but linked to our demographic surveillance by an ID number. During the community surveys written informed consent was sought from participants ≥18 years of age or the parents/guardians for children aged 10 years and below. With parental guidance, children aged over 10 and less than 18 years were asked to sign an assent form. These documents were available in Kigiriama, Kiswahili, and English. This study was approved by the Kenya Medical Research Institute Scientific Ethics Review Unit (KEMRI/SERU/CGMR-C/106/3592) and the Oxford tropical research ethics committee (OxTREC Reference: 511–18).

## Results

Between March 2018 and February 2019, 46,567 febrile patients ≥ 6 months of age sought treatment in one of the six out-patient health facilities shown in Fig 1. 18,433 were excluded because they either lived outside the study area (17,490), had been enrolled in the community survey within the last 14 days (261), were pregnant (532), or had missing mRDT results (150). Among the 28,134 febrile patients resident within the 36 EZs, 54% were female and the median age was 11 years (IQR: 4, 19 years) (Table 1). The median Euclidean distances was 1.9 km (IQR: 1.1, 2.7 km) from resident’s homestead to the facility. Among all febrile patients, 12,143 (43%) had a positive mRDT and the median age of those with a positive mRDT was 10 years (IQR: 5, 15 years). The highest TPR was among children aged 10–14 years and the lowest among adults ≥ 50 years (Table 2). TPR did not differ during the wet (42.7%) versus dry season (43.5%) (p = 0.173).

**Table 1:** Background characteristics of study participants in the health facility survey and community-based survey.

Characteristics	Health facility-based survey	Community-based survey
Total number of enumeration zones (EZ), n	36	36
Number enrolled, n	28,134	6,479
Average number of participants per EZ, (SD)	781.5 (585.1)	180.0 (161.3)
Average number of participants under-five years per EZ, (SD)	202.4 (161.0)	37.3 (32.3)
Median Euclidean distance (km) from homestead to health facility, (IQR)	1.9 (1.1, 2.7)	-
Number of non-pregnant females, n (%)	15,074 (53.6)	3,954 (61.0)
Median age in years, (IQR)	11 (4, 19)	12 (6, 24)
mRDT positivity, n (%)	12,143 (43.2)	643 (9.9)
Average number of mRDT positivity across all ages per EZ, (SD)	337.3 (330.5)	17.9 (30.6)
Average number of mRDT positivity among children under-five years per EZ, (SD)	73.0 (86.0)	3.8 (6.1)
Median age in years of mRDT positivity, (IQR)	10 (5, 15)	10 (5, 15)

EZ = enumeration zone; IQR = Interquartile Range; SD = standard deviation

**Table 2:** The relationship between health facility Test-Positivity Rate (TPR) (matched to 2 months period around each cross-sectional surveys) and community Parasite Rate (PR) or age-standardized PR_2–10 years_ stratified by age groups.

Age group	TPR (n/N, %)	PR (n/N, %)	Correlation TPR vs. community PR (95% CI)	P value	Correlation TPR vs. PR_2–10 years_ (95% CI)	P value
6–11 months	144/575 (25.0)	8/99 (8.1)	-0.11 (-0.45, 0.25)	0.540	0.27 (-0.07, 0.55)	0.119
1–4 years	1972/4830 (40.8)	128/1243 (10.3)	0.60 (0.33, 0.77)	<0.001	0.66 (0.42, 0.81)	<0.001
5–9 years	2594/4434 (58.5)	168/1278 (13.1)	0.54 (0.26, 0.74)	<0.001	0.69 (0.47, 0.83)	<0.001
10–14 years	2658/4269 (62.3)	169/1245 (13.6)	0.35 (0.02, 0.61)	0.039	0.62 (0.37, 0.79)	<0.001
15–49 years	2236/6008 (37.2)	146/2056 (7.1)	0.03 (-0.31, 0.35)	0.878	0.32 (-0.01, 0.59)	0.055
50+ years	323/1584 (20.4)	24/558 (4.3)	0.20 (-0.15, 0.51)	0.251	0.13 (-0.21, 0.44)	0.464
Overall	9927/21700 (45.8)	643/6479 (9.9)	0.63 (0.38, 0.79)	<0.001	0.62 (0.37, 0.79)	<0.001

During the four community-based prevalence surveys, 7,255 participants ≥ 6 months of age were approached for enrolment; 425 (5.9%) declined consent and 351 were excluded because they either had been enrolled in the health facility survey within the last 14 days (198), were pregnant (79) or had missing mRDT results (74). A total of 6,479 participants aged 6 months to 98 years were surveyed in the community with an average of 180 per EZ. The median age of those sampled was 12 years (IQR: 6 years, 24 years), 61% were female (Table 1) and 348 (5.8%) were febrile. The overall community PR was 9.9% (95% CI: 9.2, 10.7%) (Table 1) and was higher (27.3%) among community participants with fever. PR varied significantly across the 36 EZs (p <0.001); ranging between 0% and 41%. There were no clear patterns between seasonality and PR (9.4% in the wet vs. 10.5% in the dry; p = 0.12). The community PR was highest among children aged 10–14 years (13.6%: 95% CI: 11.7%, 15.6%) and lowest among adults aged 50 years and above (4.3%: 95% CI: 2.8%, 6.3%) (Table 2). The overall age-standardized PR_2–10 years_ was 12.7% and ranged between 0.01% and 53.2% in the 36 EZs.

**Fig 2:**
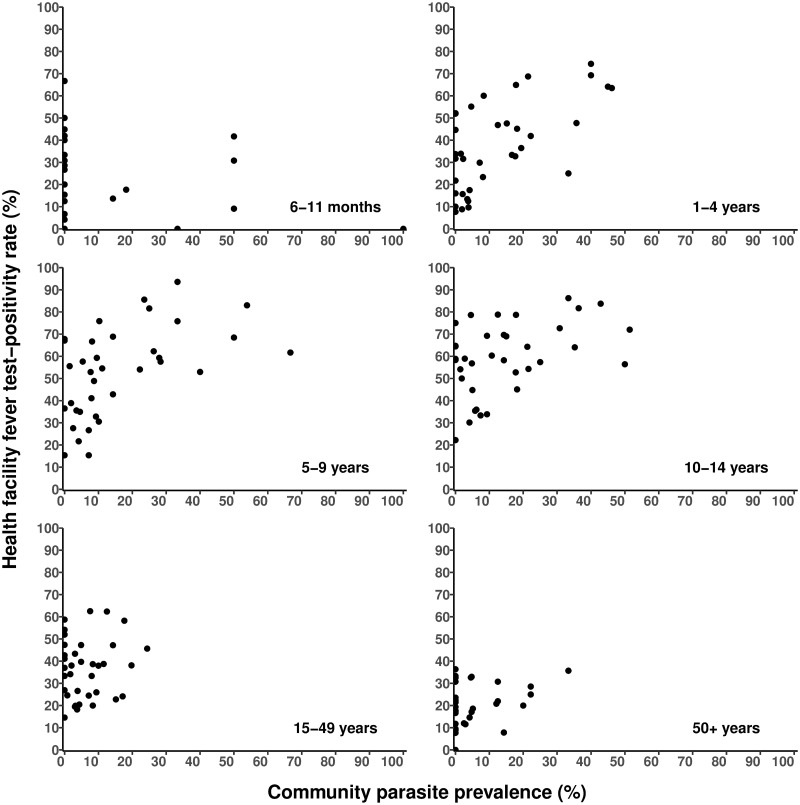
The relationship between health facility Test-Positivity Rate (TPR) (matched to 2 months period around each cross-sectional surveys) and community parasite prevalence (PR) stratified by age group.

When the health-facility data was matched to two months around the four cross-sectional surveys, there were 21,700 febrile patients aged 6 months to 95 years seen at the facilities with an average of 603 (SD = 444.6) attendees per EZ. Among this time-matched series, TPR varied significantly (p <0.001) across the 36 enumeration areas ranging between 17% and 73% and significantly differed across age groups (Table 2). The correlation between facility-based TPR and community PR across all age groups was 0.63 (95% CI: 0.38, 0.79; p <0.001) and comparable to the age-standardized PR_2–10 years_ (Table 2). The correlations were weakest among age groups 6–11 months, 15–49 years, and adults ≥ 50 years (Fig 2 and Table 2). Stronger correlations were shown in the age groups 1–4 years (*rho* = 0.60; 95% CI: 0.33, 0.77; p <0.001) and 5–9 years (*rho* = 0.54; 95% CI: 0.26, 0.74) (Fig 2 and Table 2). These comparisons were comparable across the different temporal matches of TPR data (S1 Table).

The subsequent analysis focuses on three age comparisons: 1) children aged < 5 years, as DHIS2 data is aggregated over the age groups below and above five years and household surveys measure malaria infection mostly in children aged 6 months to five years; 2) all ages, as this is also available from existing national DHIS2 platforms; and 3) TPR comparisons to age-standardized PR_2–10 years_, currently used to map malaria transmission in Africa.

**Fig 3:**
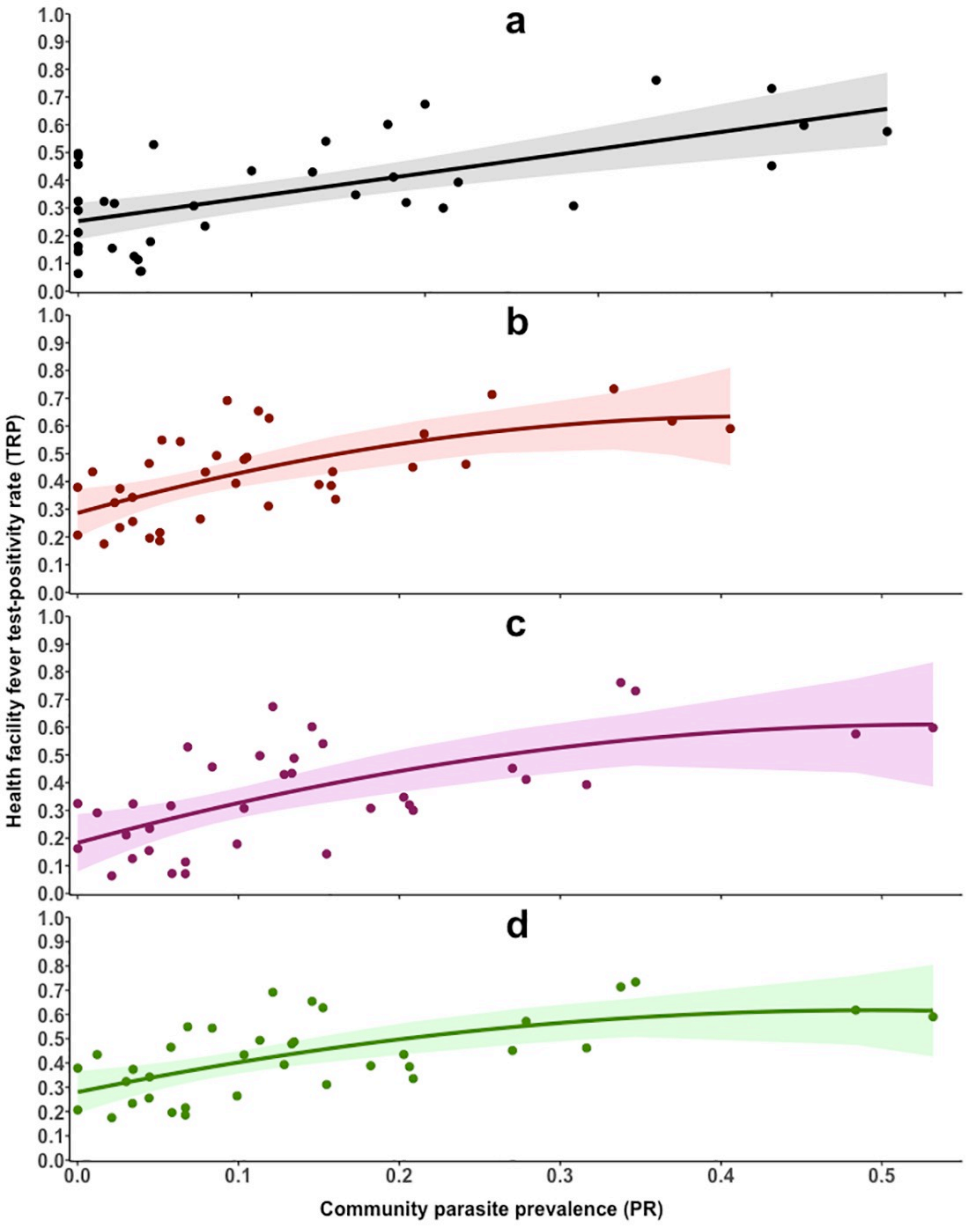
The relationship between health facility Test-Positivity Rate (TPR) and community parasite prevalence (PR). Panel A—shows the relationship between TPR_0.5–4 years_ and PR_0.5–4 years_ (black line). Panel B—shows the relationship between TPR_all ages_ and PR_all ages_ (red line). Panel C—shows the relationship between TPR_0.5–4 years_ and age-standardized PR_2–10 years_ (purple line). Panel D—shows the relationship TPR_all ages_ and age-standardized PR_2–10 years_ (green line).

To examine the functional form of the relationship between matched TPR and PR, four models were explored (S2 Table). The polynomial model of order 2 was the best fitting model, based on goodness of fit measures (RMSE, AIC and R^2^), in all comparisons, except TPR_0.5–4 years_ vs. PR_0.5–4 years_, where the linear model was the best fitting model (S2 Table and Fig 3). The selection of the age-specific models was also supported by the measures of predictive performance (MSE, MAE and MAPE) (S2 Table). Changing the temporal matches of TPR data did not alter the selection of the models (Table 3). There were significant differences in the predictive ranges of TPR using the programmatic cut-offs of PR < 5% and ≥ 30% in all the models (Table 4).

**Table 3:** Sensitivity analysis exploring the functional form of the relationship between health facility Test-Positivity Rate (TPR) and community parasite prevalence (PR) stratified by age-specific comparisons and temporal match.

Model	Coefficients			Goodness of fit		
	PR (95% CI)	PR^2^ (95% CI)	P-value	RMSE	Adjusted R^2^	AIC
**TPR**_**0.5–4 years**_ **vs PR**_**0.5–4 years**_						
TPR matched to 2 months period around cross-sectional surveys	0.87 (0.50, 1.23)	-	<0.001	0.149	0.387	-32.74
TPR matched to 4 time-periods during cross-sectional surveys	0.89 (0.54, 1.24)	-	<0.001	0.142	0.426	-36.25
TPR matched to subsequent month i.e. lagged by 1 month	0.84 (0.44, 1.25)	-	<0.001	0.165	0.324	-25.55
All time points	0.87 (0.54, 1.21)	-	<0.001	0.137	0.433	-38.80
**TPR**_**all ages**_ **vs PR**_**all ages**_						
TPR matched to 2 months period around cross-sectional surveys	1.62 (0.28, 2.96)	-1.88 (-5.42, 1.66)	<0.001	0.126	0.364	-44.00
TPR matched to 4 time-periods during cross-sectional surveys	1.56 (0.23, 2.88)	-1.82 (-5.32, 1.69)	<0.001	0.125	0.347	-44.73
TPR matched to subsequent month i.e. lagged by 1 month	1.98 (0.43, 3.52)	-2.63 (-6.70, 1.44)	<0.001	0.145	0.345	-33.9
All time points	1.74 (0.41, 3.07)	-2.19 (-5.70, 1.31)	<0.001	0.125	0.379	-44.69
**Model**	**Polynomial coefficients**			**Goodness of fit**		
**TPR**_**0.5–4 years**_ **vs PR**_**2–10 years**_						
TPR matched to 2 months period around cross-sectional surveys	1.59 (0.37, 2.80)	-1.48 (-3.97, 1.01)	<0.001	0.151	0.373	-31.04
TPR matched to 4 time-periods during cross-sectional surveys	1.61 (0.40, 2.81)	-1.58 (-4.05, 0.89)	<0.001	0.150	0.365	-31.69
TPR matched to subsequent month i.e. lagged by 1 month	1.70 (0.37, 3.02)	-1.75 (-4.47, 0.96)	<0.001	0.165	0.327	-24.77
All time points	1.66 (0.53, 2.80)	-1.68 (-4.00, 0.64)	<0.001	0.141	0.404	-36.09
**TPR**_**all ages**_ **vs PR**_**2–10 years**_						
TPR matched to 2 months period around cross-sectional surveys	1.36 (0.34, 2.39)	-1.38 (-3.47, 0.71)	<0.001	0.127	0.356	-43.55
TPR matched to 4 time-periods during cross-sectional surveys	1.32 (0.31, 2.34)	-1.37 (-3.44, 0.71)	<0.001	0.126	0.338	-44.25
TPR matched to subsequent month i.e. lagged by 1 month	1.65 (0.47, 2.83)	-1.88 (-4.29, 0.53)	<0.001	0.146	0.334	-33.34
All time points	1.45 (0.44, 2.47)	-1.56 (-3.63, 0.52)	<0.001	0.126	0.370	-44.19

Community parasite prevalence (PR); PR^2^ is a function of the polynomial equation; health facility test-positivity rate (TPR)

**Table 4:** The predictive ranges of Test Positivity Rates (TPR) for three endemicity classes of parasite prevalence (PR) stratified by age-specific comparisons.

	PR cut-offs	
	< 5%	≥ 30%
**TPR**_**0.5–4 years**_ **vs PR**_**0.5–4 years**_		
Actual predicted TPR	30%	51%
95% CI	24%, **35%**^¶^	**43%**^†^, 59%
**TPR**_**all ages**_ **vs PR**_**all ages**_		
Actual predicted TPR	35%	60%
95% CI	31%, **41%**^¶^	**52%**^†^, 69%
**TPR**_**0.5–4 years**_ **vs PR**_**2–10 years**_		
Actual predicted TPR	26%	53%
95% CI	19%, **32%**^¶^	**44%**^†^, 62%
**TPR**_**all ages**_ **vs PR**_**2–10 years**_		
Actual predicted TPR	35%	57%
95% CI	29%, **40%**^¶^	**49%**^†^, 64%

^**¶**^ For low transmission settings, the upper confidence limit of predicted TPR range was used as the conservative allocation of the maximum probable TPR as a proxy measure for PR < 5%,

^**†**^ while for high transmission settings, the lower confidence limit of the predicted TPR range was used as a conservative allocation of the minimum probable TPR as a proxy measure for PR ≥ 30%.

### Comparison among children aged < 5 years

The linear model among children aged 6 months– 4 years between the facility and community surveys suggested that only a fraction of infections in the community (PR_0.5–4 years_) developed symptoms that required treatment in an out-patient health facility (ß = 0.87; 95% CI: 0.50, 1.23; p<0.001) (Table 3 and Fig 3). The linear model estimated that a PR_0.5–4 years_ < 5% corresponded to a maximum 95% CI predicted TPR_0.5–4 years_ of < 35%; and a PR_0.5–4 years_ ≥ 30% corresponded to a minimum 95% CI predicted TPR_0.5–4 years_ of ≥ 43% (Table 4). However, the linear model performed poorly when PR_0.5–4 years_ was >85% leading to predicted values for TPR_0.5–4 years_ greater than 100%, if the linear model holds true outside of the observed values.

### Comparison across all ages

A polynomial model was used for all age-groups from facility and community surveys (Table 3 and Fig 3). The model signified slightly larger changes in the predicted TPR_all ages_ when PR_all ages_ was below 10%, but smaller changes in the predicted TPR_all ages_ when PR_all ages_ was above 30% (Fig 3). The polynomial model estimated that a PR_all ages_ < 5% corresponded to a maximum 95% CI predicted TPR_all ages_ of approximately < 41%; and a PR_all ages_ ≥ 30% corresponded to a minimum 95% CI predicted TPR_all ages_ ≥ 52% (Table 4). Beyond a PR_all ages_ of 45%, the polynomial model predictions of TPR_all ages_ saturates, if the polynomial model holds true outside of the observed values.

### Comparison to age-standardized PR_2–10 years_

The analysis was repeated using age-standardized PR_2–10 year_ data. Here a polynomial model best described the relationships between TPR_0.5–4 years_ vs. PR_2–10 year_ and TPR_all ages_ vs. PR_2–10 year_ (Table 3 and Fig 3). The polynomial model estimates that an age-standardized PR_2–10 year_ < 5% corresponded to a maximum 95% CI predicted TPR_0.5–4 years_ of < 32%, which were lower than that estimated using the linear model. The age-standardized PR_2–10 year_ ≥ 30% corresponded to a similar minimum 95% CI predicted TPR_0.5–4 years_ of ≥ 44%. When the standardized PR_2–10 year_ was beyond 65%, the polynomial model saturated. For TPR_all ages_ the predictive accuracies of the endemicity classes of the age-standardized PR_2–10 year_ were similar to those predicted using the actual PR_all ages_ data (Table 4).

In summary, although the polynomial model might not be discriminatory enough it was the best fitting model for the data that described the algebraic relationship between TPR and PR. In this setting, a TPR of ≥ 49% in all age groups would correspond to a PR_2–10 years_ of ≥ 30%, while a TPR_all ages_ of < 40% would correspond to an age-standardized PR_2–10 years_ of < 5%.

## Discussion

The utility of malaria surveillance passively collected at health facilities as a surrogate to community infection prevalence remains poorly defined. To characterise this relationship, 36 paired facility-based TPR and community PR were examined over a 12-month period on the Kenyan coast. In the present study less than 10% of people were harbouring malaria infections at any point in time, however, more than 40% of those with a fever attending a facility had an infection. In this low-moderate transmission setting, fever might be a good predictor of infection. During the community-based surveys, 22% of children aged 6 months—4 years reporting fever were mRDT positive, compared to 9% of afebrile children, differences described in household surveys across Africa [53].

In this study, there was a strong positive correlation between facility-based TPR and community PR reported across all ages. The association between TPR and PR was higher in children aged 6 months– 14 years than in adults aged ≥15 years since in this low-moderate transmission setting, children were more likely to become symptomatic leading to prompt care-seeking. However, the association could also represent opportunistically detected malaria infections in children, meaning, fevers seen in the facilities might not be causally related to malaria infection. A direct non-linear, polynomial relationship was observed between the relative change in TPR and changes in the PR, suggesting a statistical relationship between TPR as a proxy for traditional community-based measures of malaria transmission. However, polynomial model fitting of routine data might not lend itself easily to programmatic use for malaria stratification by the NMCP, where simpler cut-offs are required.

NMCPs must make strategic policy decisions for intervention based on data to either sustain and accelerate existing disease control efforts or migrate to more efficient systems of case-detection on a pathway to elimination [54]. Decisions to-date have relied heavily on interpolated, infrequent and incomplete community-based infection prevalence maps. There are no definitive guidelines on the cut-offs based on PR in relation to selection of specific interventions, except for Seasonal Malaria Chemoprevention [55]. However, several countries have elected to use pragmatic cut-offs of <5% PR to consider revising policies on vector control and prevention of malaria in pregnancy; while identifying areas ≥ 30% PR that demand improved coverage of all prevention strategies and increased sub-national malaria control investment [30, 51]. Within the study area, a maximum probable TPR of < 40% would correspond to areas with a PR of < 5%, while a corresponding minimum probable TPR of approximately ≥49% would identify high transmission areas (PR ≥ 30%) in need of addition malaria prevention. These two predictive ranges of TPR, defined through testing all fevers, were significantly different in all the models, however, the actual differences in the upper and lower bounds of the conservative values 40% versus 49% TPR might not provide NMCPs adequate discriminatory power when using data collected under routine conditions. Electing to migrate from one vector control strategy to another to promote sub-county policy decisions on a pathway to elimination, based on these narrow discriminatory cut-offs seems unlikely. Larger, more spatially diverse data series using routinely collected TPR matched to community-based infection prevalence data are required to explore the more practical implications of using TPR as a replacement for community PR. The aim here was to show that a statistical relationship does exist using carefully controlled data.

The relationships between TPR and PR shown here are consistent with other studies undertaken using similar designs. In three separate transmission settings in western Kenya, the correlation between malaria positivity rate among suspected patients from the health facilities and asymptomatic malaria positivity among school children was 0.78, 0.61, and -0.039 in the low, moderate, and high transmission settings, respectively [43]. As with the present study, the weakest correlation was also reported for TPR among individuals aged ≥ 15 years in the moderate (*rho* = 0.32) and high transmission areas (*rho* = 0.01) [43]. A much earlier study, outside of Africa, in Punjab, Pakistan, found a stronger positive correlation (*rho* = 0.97) between clinic slide positivity data and community survey data from four villages compared at three different periods of observation [42].

Routinely collected TPR has several advantages as a surrogate measure of malaria transmission. There are obvious opportunity costs of using routine, rather than expensive survey data; data are spatially cosmopolitan rather than opportunistically sampled; available at continuous temporal resolutions; and provide granular data at district levels for district level decision making. In the present study, national guidelines [48] on test-treat among all age groups were followed, to this end all fevers were tested, mRDTs were supplied by the research team and careful documentation of all events formed the basis of the study. Under normal health facility conditions, the reality is likely to be very different. Studies have shown that not all fevers presenting to clinics are tested and reporting rates are often incomplete [23, 56–59]. Although TPR has been shown to be a useful measure in the reflection of infection transmission dynamics in the communities [42, 43], there are several important considerations. First, despite its low cost and simplicity, the use of TPR is hugely dependent on coverage, completeness, quality of information [27, 60, 61], and may be affected by health seeking behaviors, and diagnostic test utilization [3]. Variations in the testing rates have been associated with levels of endemicity, staffing or workload, inadequate training and lack of supervision of health care workers, shortages and stock-outs of mRDTs, and patient-level factors [23]. In Kenya, there is increasing evidence that coverage, completeness, quality of routine reliable malaria information remains woefully inadequate [60–62]. These inadequacies are not insurmountable. They represent a reality that requires health systems investments to change. Moreover, surveillance data is a key pillar of intervention necessary for future national malaria control in Africa [63].

Although an out-of-sample validation was performed, the confidence in the generalizability of the results is reinforced if it is validated in an external population. Furthermore, caution should be used in interpreting the function of best-fit regression. Noise in the estimation of either variable will lead to not just uncertainty in the estimate, but also to a slope that is biased towards zero due to the effect of regression dilution bias. Noise can be introduced by completely random errors (minimized by sample size) and other errors introduced by heterogeneity in transmission. It is possible that larger datasets would lead to the estimation of a steeper function linking PR to TPR, which would then lead to a more favourable impression of the utility of TPR in determining endemicity.

## Conclusion

Health facility-based surveillance with indicators like TPR remains an attractive measure which might be a crude reflection of transmission dynamics while at the same time, they are more operationally attractive compared with community-based surveys in terms of time and cost. However, a better understanding is required of the biological, clinical, social and epidemiological relationships between malaria infection and fever across all ages, and all health systems. Importantly, these studies must be undertaken across a wide range of endemicities to develop a more pragmatic usable criteria for NMCPs to use effectively in a stratified response to malaria control.

## Supporting information

S1 Table The correlation between health facility fever Test-Positivity Rate (TPR) and community Parasite Rate (PR) stratified by age group and varying the intervals of TPR as described in the methods section. (DOCX)

S2 Table Regression models of diagnostic Test Positivity Rates (TPR) as predictors of parasite prevalence (PR) stratified by age. (DOCX)

S1 File (PDF)
