# Supplementary material for: The relationship between facility-based malaria test positivity rate and community-based parasite prevalence
Source: PLoS One. 2020 Oct 7;15(10):e0240058. doi: 10.1371/journal.pone.0240058 (PMC7540858; doi:10.1371/journal.pone.0240058)
Supplement: S1 Table — (DOCX) [file pone.0240058.s001.docx]

**S1 Table:** The correlation between health facility fever test-positivity rate (TPR) and community parasite rate (PR) stratified by age group and varying the intervals of TPR as described in the methods section

|  | **All time points** | | **Matched to 4 time-periods during cross-sectional surveys** | | **Matched to subsequent month i.e. lagged by 1 month** | | **Matched to 2 months period around cross-sectional surveys** | |
| --- | --- | --- | --- | --- | --- | --- | --- | --- |
| **Age group** | **Correlation (95% CI)** | **P value** | **Correlation (95% CI)** | **P value** | **Correlation (95% CI)** | **P value** | **Correlation (95% CI)** | **P value** |
| 6 - 11 months | -0.10  (-0.44, 0.26) | 0.581 | -0.16  (-0.48, 0.21) | 0.399 | 0.05  (-0.32, 0.41) | 0.793 | -0.11  (-0.45, 0.25) | 0.540 |
| 1 - 4 years | 0.62  (0.37, 0.79) | <0.001 | 0.64  (0.40, 0.80) | <0.001 | 0.50  (0.21, 0.71) | 0.002 | 0.60  (0.33, 0.77) | <0.001 |
| 5 - 9 years | 0.56  (0.28, 0.75) | <0.001 | 0.49  (0.20, 0.71) | 0.002 | 0.54  (0.26, 0.74) | <0.001 | 0.54  (0.26, 0.74) | <0.001 |
| 10 - 14 years | 0.37  (0.05, 0.62) | 0.026 | 0.32  (-0.01, 0.59) | 0.056 | 0.33  (-0.003, 0.59) | 0.053 | 0.35  (0.02, 0.61) | 0.039 |
| 15 - 49 years | 0.06  (-0.27, 0.38) | 0.721 | 0.12  (-0.21, 0.44) | 0.470 | 0.13  (-0.21, 0.44) | 0.445 | 0.03  (-0.31, 0.35) | 0.878 |
| 50+ years | 0.16  (-0.19, 0.48) | 0.359 | 0.16  (-0.19, 0.48) | 0.358 | 0.12  (-0.22, 0.44) | 0.487 | 0.20  (-0.15, 0.51) | 0.251 |
| All ages | 0.65  (0.41, 081) | <0.001 | 0.61  (0.35, 0.78) | <0.001 | 0.60  (0.33, 0.77) | <0.001 | 0.63  (0.38, 0.79) | <0.001 |
