# Supplementary material for: The relationship between facility-based malaria test positivity rate and community-based parasite prevalence
Source: PLoS One. 2020 Oct 7;15(10):e0240058. doi: 10.1371/journal.pone.0240058 (PMC7540858; doi:10.1371/journal.pone.0240058)
Supplement: S2 Table — (DOCX) [file pone.0240058.s002.docx]

**S2 Table:** Regression models of diagnostic test positivity rates (TPR) as predictors of parasite prevalence (PR) stratified by age

| **Model** | **Formula** | **RMSE** | **Adjusted R^2^** | **AIC** | ***rho*^¶^** | **MAE** | **MSE** | **MAPE** |
| --- | --- | --- | --- | --- | --- | --- | --- | --- |
| **TPR_0.5-4 years_ vs. PR_0.5-4 years_** | | | |  |  |  |  |  |
| **Linear** | **y = b0 + b1x** | **0.149** | **0.387** | **-32.74** | **0.61** | **0.13** | **0.02** | **0.50** |
| Exponential | y = b0 * b1^x^ | 0.561 | 0.298 | 62.46 | 0.61 | 0.14 | 0.03 | 0.47 |
| Cubic | y = b0 + b1x + b2x^2^ + b3x^3^ | 0.152 | 0.364 | -29.59 | 0.55 | 0.14 | 0.03 | 0.55 |
| Polynomial order 2 | y = b0 + b1x + b2x^2^ | 0.151 | 0.372 | -30.97 | 0.6 | 0.13 | 0.02 | 0.52 |
| **TPR_all ages_ vs. PR_all ages_** | | | | |  |  |  |  |
| Linear | y = b0 + b1x | 0.127 | 0.360 | -44.74 | 0.62 | 0.11 | 0.01 | 0.28 |
| Exponential | y = b0 * b1^x^ | 0.333 | 0.324 | 24.89 | 0.62 | 0.11 | 0.02 | 0.28 |
| Cubic | y = b0 + b1x + b2x^2^ + b3x^3^ | 0.128 | 0.344 | -42.02 | 0.56 | 0.11 | 0.02 | 0.29 |
| **Polynomial order 2** | **y = b0 + b1x + b2x^2^** | **0.126** | **0.364** | **-44.00** | **0.60** | **0.11** | **0.02** | **0.28** |
| **TPR_0.5-4 years_ vs. PR_2-10 years_** | |  |  |  |  |  |  |  |
| Linear | y = b0 + b1x | 0.152 | 0.365 | -31.49 | 0.62 | 0.13 | 0.02 | 0.54 |
| Exponential | y = b0 * b1^x^ | 0.564 | 0.290 | 62.87 | 0.59 | 0.15 | 0.03 | 0.51 |
| Cubic | y = b0 + b1x + b2x^2^ + b3x^3^ | 0.153 | 0.354 | -29.04 | 0.59 | 0.13 | 0.02 | 0.56 |
| **Polynomial order 2** | **y = b0 + b1x + b2x^2^** | **0.151** | **0.373** | **-31.04** | **0.61** | **0.13** | **0.02** | **0.53** |
| **TPR_all ages_ vs. PR_2-10 years_** | | |  |  |  |  |  |  |
| Linear | y = b0 + b1x | 0.128 | 0.341 | -43.65 | 0.59 | 0.11 | 0.02 | 0.29 |
| Exponential | y = b0 * b1^x^ | 0.336 | 0.313 | 25.50 | 0.59 | 0.12 | 0.02 | 0.29 |
| Cubic | y = b0 + b1x + b2x^2^ + b3x^3^ | 0.129 | 0.336 | -41.56 | 0.56 | 0.11 | 0.02 | 0.30 |
| **Polynomial order 2** | **y = b0 + b1x + b2x^2^** | **0.127** | **0.356** | **-43.55** | **0.58** | **0.11** | **0.02** | **0.29** |

**^¶^ *rho* =** Correlation between actual and predicted TPR; root mean square error (RMSE); mean absolute error (MAE); mean square error (MSE); mean absolute percentage error (MAPE)

**Footnote**: The linear regression model in some age groupings predicted values for TPR that were outside the (0, 1) interval. For example, a standardized PR_2-10 years_ beyond 85% corresponds to a predicted TPR_0.5-4 years_ >100%. Similarly, a community PR_all ages_ beyond 72% corresponds to a predicted TPR_all ages_ >100%.
