# Supplementary material for: The relationship between facility-based malaria test positivity rate and community-based parasite prevalence
Source: PLoS One. 2020 Oct 7;15(10):e0240058. doi: 10.1371/journal.pone.0240058 (PMC7540858; doi:10.1371/journal.pone.0240058)
Supplement: S1 File — (PDF) [file pone.0240058.s003.pdf]

S1 Table: The correlation between health facility fever test-positivity rate (TPR) and community parasite rate (PR) stratified by age group and varying the intervals of TPR as described in the methods section

	All time points		Matched to 4 time-periods during cross-sectional surveys		Matched to subsequent month i.e. lagged by 1 month		Matched to 2 months period around cross-sectional surveys	
**Age group**	**Correlation (95% CI)**	**P value**	**Correlation (95% CI)**	**P value**	**Correlation (95% CI)**	**P value**	**Correlation (95% CI)**	**P value**
6 - 11 months	-0.10 (-0.44, 0.26)	0.581	-0.16 (-0.48, 0.21)	0.399	0.05 (-0.32, 0.41)	0.793	-0.11 (-0.45, 0.25)	0.540
1 - 4 years	0.62 (0.37, 0.79)	<0.001	0.64 (0.40, 0.80)	<0.001	0.50 (0.21, 0.71)	0.002	0.60 (0.33, 0.77)	<0.001
5 - 9 years	0.56 (0.28, 0.75)	<0.001	0.49 (0.20, 0.71)	0.002	0.54 (0.26, 0.74)	<0.001	0.54 (0.26, 0.74)	<0.001
10 - 14 years	0.37 (0.05, 0.62)	0.026	0.32 (-0.01, 0.59)	0.056	0.33 (-0.003, 0.59)	0.053	0.35 (0.02, 0.61)	0.039
15 - 49 years	0.06 (-0.27, 0.38)	0.721	0.12 (-0.21, 0.44)	0.470	0.13 (-0.21, 0.44)	0.445	0.03 (-0.31, 0.35)	0.878
50+ years	0.16 (-0.19, 0.48)	0.359	0.16 (-0.19, 0.48)	0.358	0.12 (-0.22, 0.44)	0.487	0.20 (-0.15, 0.51)	0.251
All ages	0.65 (0.41, 081)	<0.001	0.61 (0.35, 0.78)	<0.001	0.60 (0.33, 0.77)	<0.001	0.63 (0.38, 0.79)	<0.001

S2 Table: Regression models of diagnostic test positivity rates (TPR) as predictors of parasite prevalence (PR) stratified by age

Model	Formula	RMSE	Adjusted R^2	AIC	*rho*¶	MAE	MSE	MAPE
**$TPR_{0.5\text{-}4\ years}$ vs. $PR_{0.5\text{-}4\ years}$**								
**Linear**	**$y = b0 + b1x$**	**0.149**	**0.387**	**-32.74**	**0.61**	**0.13**	**0.02**	**0.50**
Exponential	$y = b0 * b1^x$	0.561	0.298	62.46	0.61	0.14	0.03	0.47
Cubic	$y = b0 + b1x + b2x^2 + b3x^3$	0.152	0.364	-29.59	0.55	0.14	0.03	0.55
Polynomial order 2	$y = b0 + b1x + b2x^2$	0.151	0.372	-30.97	0.6	0.13	0.02	0.52
**$TPR_{all\ ages}$ vs. $PR_{all\ ages}$**								
Linear	$y = b0 + b1x$	0.127	0.360	-44.74	0.62	0.11	0.01	0.28
Exponential	$y = b0 * b1^x$	0.333	0.324	24.89	0.62	0.11	0.02	0.28
Cubic	$y = b0 + b1x + b2x^2 + b3x^3$	0.128	0.344	-42.02	0.56	0.11	0.02	0.29
**Polynomial order 2**	**$y = b0 + b1x + b2x^2$**	**0.126**	**0.364**	**-44.00**	**0.60**	**0.11**	**0.02**	**0.28**
**$TPR_{0.5\text{-}4\ years}$ vs. $PR_{2\text{-}10\ years}$**								
Linear	$y = b0 + b1x$	0.152	0.365	-31.49	0.62	0.13	0.02	0.54
Exponential	$y = b0 * b1^x$	0.564	0.290	62.87	0.59	0.15	0.03	0.51
Cubic	$y = b0 + b1x + b2x^2 + b3x^3$	0.153	0.354	-29.04	0.59	0.13	0.02	0.56
**Polynomial order 2**	**$y = b0 + b1x + b2x^2$**	**0.151**	**0.373**	**-31.04**	**0.61**	**0.13**	**0.02**	**0.53**
**$TPR_{all\ ages}$ vs. $PR_{2\text{-}10\ years}$**								
Linear	$y = b0 + b1x$	0.128	0.341	-43.65	0.59	0.11	0.02	0.29
Exponential	$y = b0 * b1^x$	0.336	0.313	25.50	0.59	0.12	0.02	0.29
Cubic	$y = b0 + b1x + b2x^2 + b3x^3$	0.129	0.336	-41.56	0.56	0.11	0.02	0.30
**Polynomial order 2**	**$y = b0 + b1x + b2x^2$**	**0.127**	**0.356**	**-43.55**	**0.58**	**0.11**	**0.02**	**0.29**

¶ ***rho* =** Correlation between actual and predicted TPR; root mean square error (RMSE); mean absolute error (MAE); mean square error (MSE); mean absolute percentage error (MAPE)

Footnote: The linear regression model in some age groupings predicted values for TPR that were outside the (0, 1) interval. For example, a standardized $PR_{2\text{-}10\ years}$ beyond 85% corresponds to a predicted $TPR_{0.5\text{-}4\ years}$ >100%. Similarly, a community $PR_{all\ ages}$ beyond 72% corresponds to a predicted $TPR_{all\ ages}$ >100%.
